# Giant Chondromyxoid Fibroma Associated With Epileptic Seizures: A Case Report

**DOI:** 10.7759/cureus.70950

**Published:** 2024-10-06

**Authors:** Corneliu Toader, Mugurel Petrinel Radoi, Luca-Andrei Glavan, Razvan-Adrian Covache-Busuioc, Milena Monica Ilie, Mihail Popa, Adrian Dumitru

**Affiliations:** 1 Department of Neurosurgery, Carol Davila University of Medicine and Pharmacy, Bucharest, ROU; 2 Department of Vascular Neurosurgery, National Institute of Neurology and Neurovascular Diseases, Bucharest, ROU; 3 Department of Pathology, Carol Davila University of Medicine and Pharmacy, Bucharest, ROU

**Keywords:** chondromyxoid fibroma, epileptic seizures, neurosurgery, skull base, subtotal resection

## Abstract

This case report presents an exceedingly rare instance of skull base chondromyxoid fibroma (CMF) managed surgically. Chondromyxoid fibromas are very rare tumors (<1% of benign bone tumors), the occurrence in the skull area being even more rare. The location of tumors at the skull base makes their surgical resection extremely challenging, usually resulting in subtotal resection (STR). One aspect that makes this case stand out is its unique clinical presentation, particularly the presence of epileptic seizures. Patients suffering from skull base CMFs must receive regular follow-up exams in order to track disease progress, maintain quality of life, and prevent further complications.

## Introduction

Chondromyxoid fibromas (CMFs) are rare, benign cartilaginous neoplasms, comprising less than 1% of all bone tumors. These benign tumors originate from cartilage-forming tissue and are distinguished by a mixture of chondroid, myxoid, and fibrous tissue components, which are arranged in a pseudolobulated architecture [1,2].

Chondromyxoid fibromas can affect both males and females, though some studies indicate a slight male predominance, with reported ratios of approximately 2:1 [3]. Chondromyxoid fibromas usually arise in the metaphyseal region of long bones near the knee, often found on either the proximal tibia (near the knee), distal femur (near the knee), bones of the foot or pelvis/sacrum, as well as hand/foot bones and occasionally the skull base or mandible [4,5]. They typically arise before the age of 30, with the highest incidence occurring between 10 and 30 years. Chondromyxoid fibromas typically manifest early, often being diagnosed before the age of 30, with the highest incidence occurring between 20 and 30 years [6, 7].

Chromosomal studies have noted associations with certain abnormalities, including clonal rearrangements of chromosome 6 and upregulation of GRM1 genes in some instances, suggesting an underlying genetic component to these disorders [8].

Chondromyxoid fibromas of the skull typically exhibit slow, steady growth, with local pain often being the presenting symptom. Although neurological deficits are relatively uncommon among them, they may occur as the tumor expands and encompasses nearby structures, including cranial nerves [9]. The particularity of this case lies in the extremely rare location of the chondromyxoid tumor, its large size, and the achievement of an aggressive surgical resection.

## Case presentation

A 37-year-old female presented to our neurosurgical department following a generalized seizure that began three days prior to her admission. A cerebral CT scan revealed an intracranial extra-axial tumor in the right cerebral hemisphere. She was referred to our service for further investigation and specialized therapeutic management. Neurological examination revealed the patient to be conscious but confused, with slowed speech and thought processes, a Glasgow Coma Scale (GCS) score of 13, language disturbances, and mild left hemiparesis (3/5 on the Medical Research Council (MRC) grading system).

**Figure 1:**
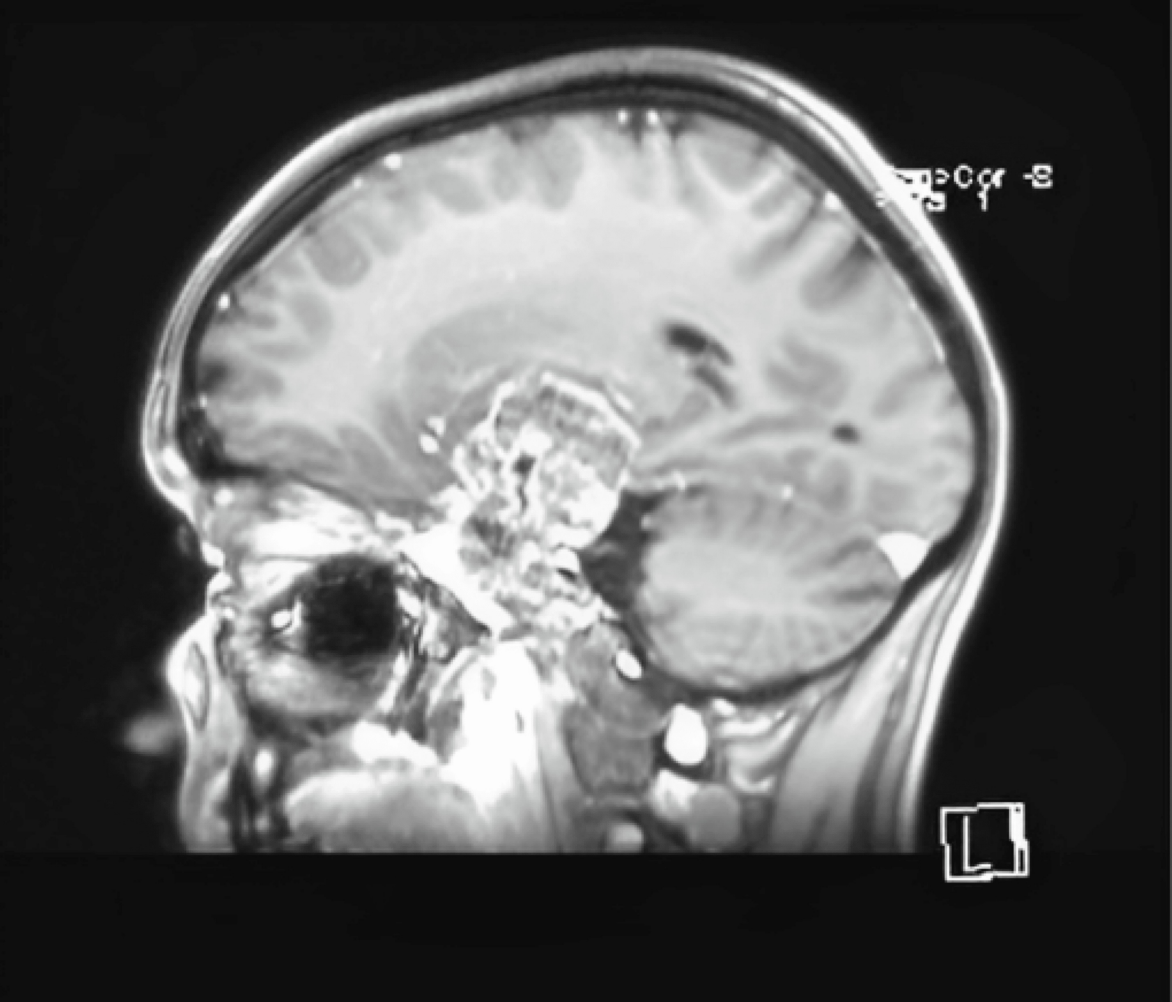
Sagittal section of the MRI examination The MRI shows a voluminous, expansive, multilobulated process in the right temporal lobe with an infratentorial component that engulfs both the middle cerebral artery and the apex of the basilar artery.

**Figure 2:**
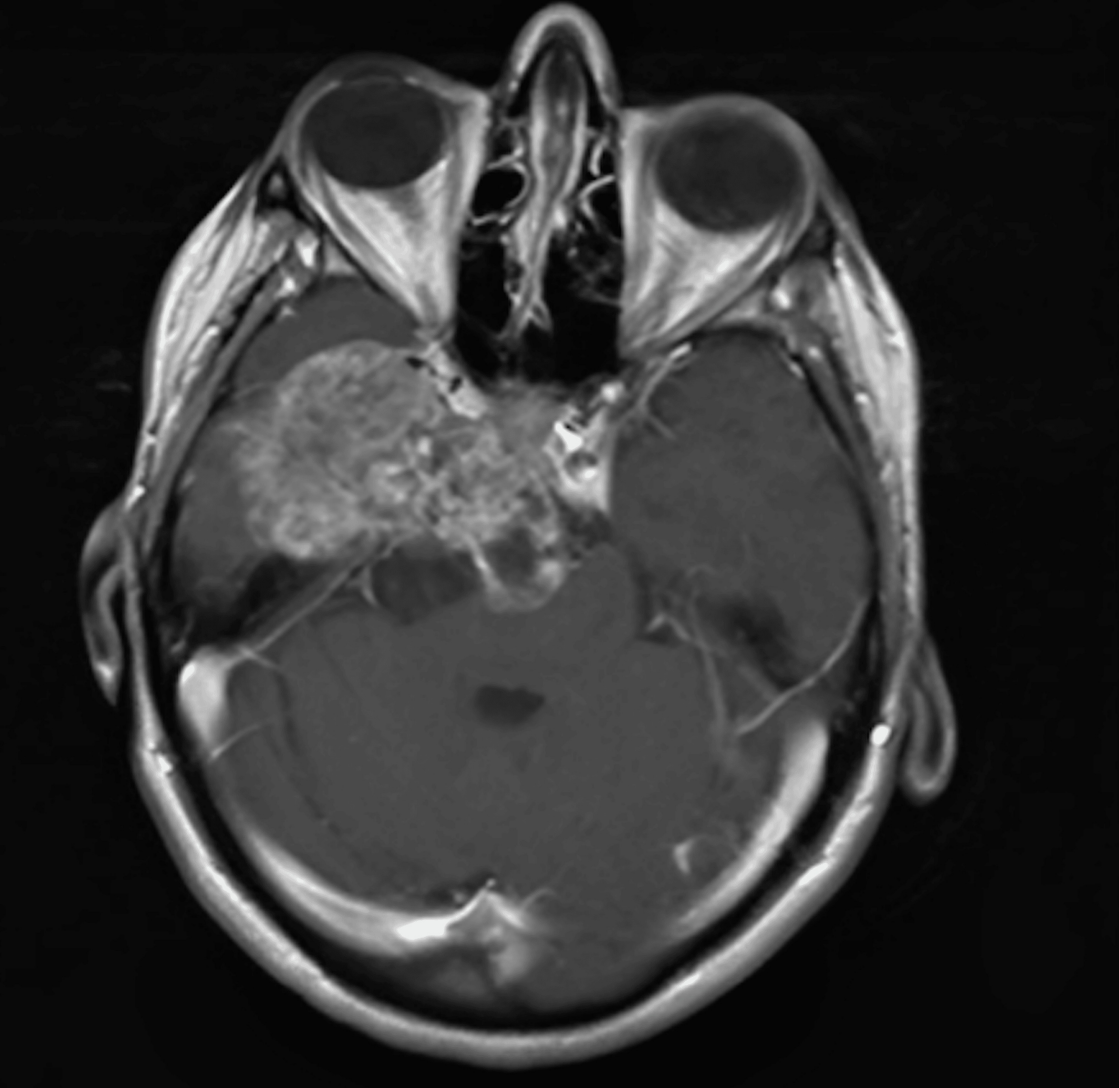
Transverse section of the MRI An infiltrative component is observed in the right half of the clivus, the right half of the sella turcica, and the cavernous sinus.

Magnetic resonance imaging with and without contrast showed a voluminous, extraneural, expansive, multilobulated process with a hyperintense T2 signal and heterogeneous gadolinium enhancement, measuring approximately 82x40x52 mm. The tumor was multicompartmental, including a component in the right temporal pericerebral area, exerting a mass effect on the adjacent cerebral parenchyma. Another component was located in the posterior fossa within the prepontine and interpeduncular cisterns, exerting a mass effect on the brainstem and engulfing the apex of the basilar artery, a suprasellar component with a mass effect on the hypothalamus, the right half of the sella turcica, and the cavernous sinus. Additionally, a suprasellar component exerted a mass effect on the hypothalamus, and an infiltrative component was observed in the right half of the clivus, the right half of the sella turcica, and the cavernous sinus. Intratumoral areas revealed degradation products of hemoglobin in subacute and chronic stages, along with a network of neovascularization. The intracavernous and supraclinoid segments of the right internal carotid artery were displaced anteriorly and laterally by the expansile process. The septum pellucidum was displaced approximately 10 mm to the left, with minimal dilation of the occipital horns of the lateral ventricles bilaterally and the frontal horn of the left lateral ventricle, accompanied by minimal transependymal cerebrospinal fluid resorption edema. No other signal abnormalities were noted in the infratentorial or supratentorial cerebral parenchyma. There was a slight anterior protrusion of the right eyeball (Figures 1, 2).

**Figure 3:**
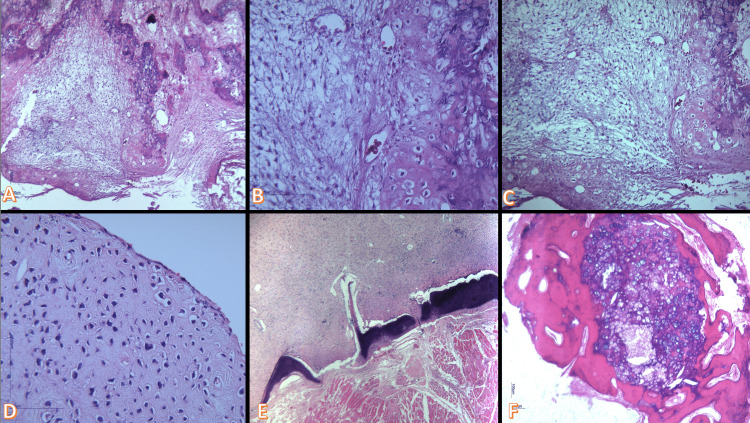
(A) Lobules have stellate cells in a myxoid background and reside in lacunae in chondroid areas (H&E 10X); (B) Lobules have stellate cells in a myxoid background and reside in lacunae in chondroid areas (H&E 40x); (C) Myxoid to chondroid stroma, representing various stages of cartilaginous development (H&E 20x); (D) Paucicellular chondroid proliferation consisting of relatively monomorphic chondrocytic type cells mixed with fibroblastic type cells with a stellate or spindle shape appearance (H&E 40x); (E) Slightly lobular chondroid tumor formation showing on the periphery a fragment of sclerotic bone tissue and skeletal muscle fibers (H&E 4x); (F) Come chondroid areas are sequestered in compact bone tissue with areas of osteosclerosis (H&E 4x)

**Figure 4:**
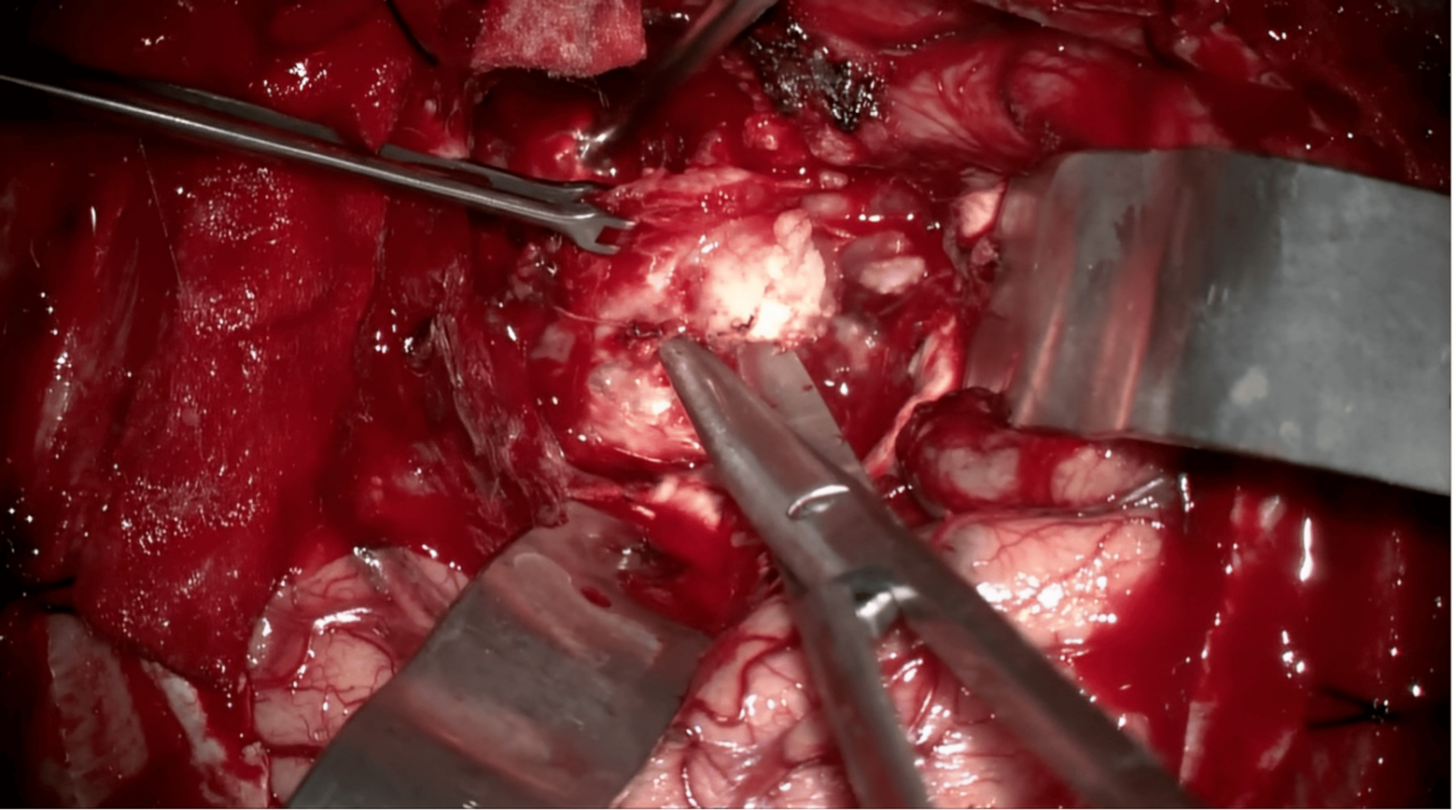
Surgical resection of the tumor Sub-total resection of the tumoral mass

Surgical intervention was performed, under general anesthesia, involving a right frontotemporal craniotomy and subtotal resection (STR) of the extra-axial tumor (Figure 3). Subtotal resection was performed because the tumor engulfed the middle cerebral artery and its bifurcation, a part of the basilar artery, the right cavernous sinus, and the sella turcica. Therefore, the risk of hemorrhage was great, and STR ensured no such complications (Figure 4). The postoperative course was slowly favorable, with no new neurological deficits. Postoperative follow-up CT scans reveal subtotal removal of the tumor without hemorrhagic complications. Follow-up cranial CT scans indicated no immediate postoperative complications. At discharge, the patient was conscious, cooperative with an improved left motor deficit (4/5 MRC), and able to walk without support.

**Figure 5:**
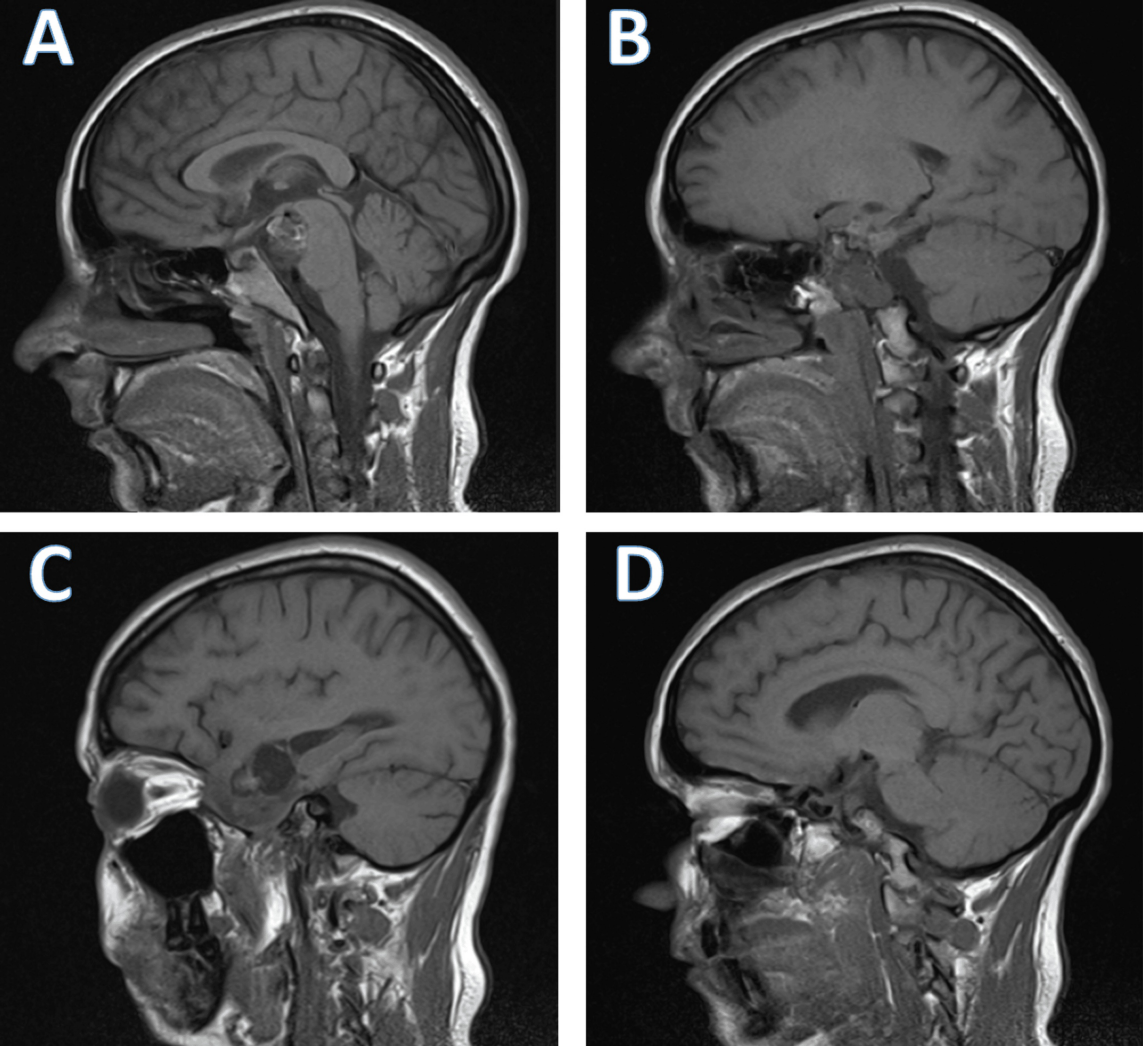
MRI with contrast showed a minimal area of tumor recurrence in the right basal frontotemporal region, without mass effect or indication for surgery.

At the three-month follow-up, the patient's condition showed significant neurological improvement. She presented with non-systematized balance disturbances, but no motor deficits. Right eye exophthalmos and chemosis which existed before surgery, were still present. Cerebral MRI with gadolinium enhancement showed residual tumors in the right basal temporal region and intrapeduncular and prepontine cisterns (Figure 5). There were no indications for reoperation.

**Figure 6:**
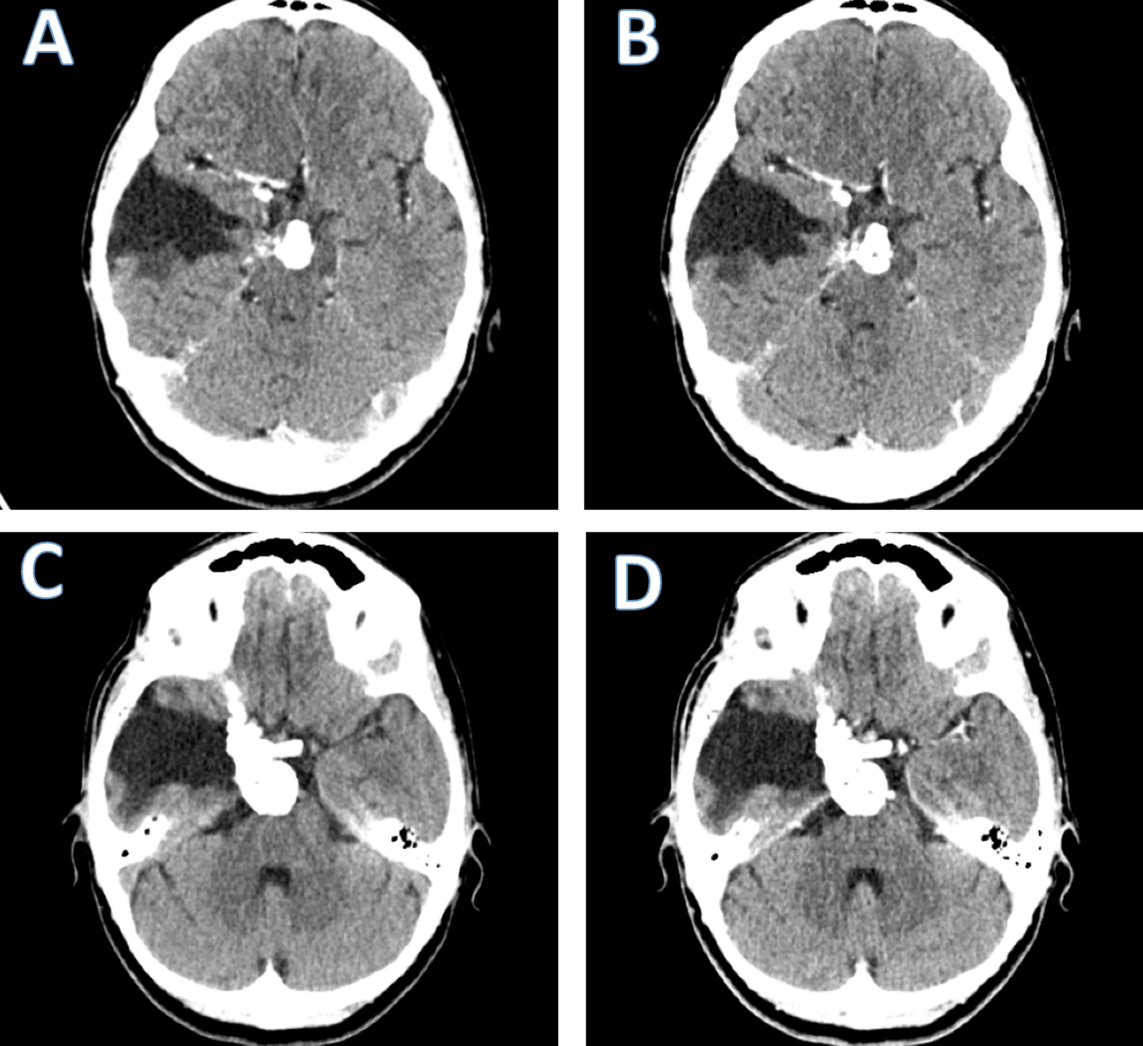
A cranial CT scan, both native and with contrast, demonstrated minimal area of tumor recurrence in the right basal frontotemporal region, without mass effect or surgical indication.

The patient's neurological condition remained favorable at the 12-month follow-up, exhibiting no focal neurologic deficits. Right eye exophthalmos remained unchanged (Figure 6).

## Discussion

To our knowledge, this is the only reported case in the literature where epileptic seizures were a significant neurological manifestation of CMF. While seizures have been commonly associated with intracranial tumors such as gliomas and meningiomas due to their mass effect and tendency to cause peritumoral edema, their occurrence in the context of CMF is rare. In a study by Kadom et al. on frontal bone CMF, no seizures were reported, despite the tumor's large size and proximity to brain structures [10]. This suggests that the specific location and pattern of involvement in our case, particularly the tumor's engulfment of the apex of the basal artery and compression of the right posterior cerebral artery, may play a unique role in triggering seizure activity. Vascular compression has been documented in other skull base tumors as a potential cause of ischemia and subsequent metabolic disturbances, which can lower the threshold for seizure generation [11,12]. This pattern of vascular involvement contrasts with previously reported cases of CMF where the mass effect was the primary neurological concern without seizure manifestation [11,13].

Moreover, the hypothalamic involvement in our case provides an additional layer of complexity. The hypothalamus plays a key role in regulating autonomic functions and maintaining hormonal balance. Compression of the hypothalamus can lead to disruptions in these functions, influencing seizure thresholds, as has been suggested in studies on hypothalamic gliomas and craniopharyngiomas [14,15]. These tumors, similar to the one in our case, exert a mass effect on the hypothalamus, leading to seizure activity due to altered metabolic and neurohormonal dynamics. However, in contrast to such tumors, where hypothalamic involvement is a common feature, involvement in CMF is exceedingly rare, making our case stand out.

Additionally, compression of the clivus, sella turcica, and cavernous sinus, as seen in our patient, can lead to a variety of neurological symptoms, including seizures, due to the proximity to critical neurovascular structures. The mass effect causing a midline shift of 10 mm is another contributing factor to the development of seizures. Midline shift is frequently associated with raised intracranial pressure and is often implicated in seizure disorders in patients with brain tumors, even in cases where the primary pathology is not inherently epileptogenic [16,17].

In comparison, Xu et al. also reported vascular involvement in skull base CMF cases, where mass effect contributed to neurological symptoms, though seizures were not explicitly noted [11]. The absence of seizures in their cases may reflect a difference in tumor location or extent of vascular compression, suggesting that the precise anatomical involvement is a crucial determinant in seizure development.

Our case also emphasizes the importance of gross total resection (GTR) for CMF, especially in cases with neurological manifestations. Gross total resection has been associated with improved outcomes in terms of symptom reduction and quality of life, as seen in other reports of skull base tumors [14,18]. However, our patient, who underwent STR, experienced early tumor recurrence, further underscoring the risk associated with incomplete tumor removal. This aligns with findings in the literature, where recurrence rates for CMF, particularly in the skull base, are estimated to be around 17%-20% [10, 13].

The role of postoperative radiotherapy for CMF remains controversial. Some studies suggest that radiotherapy may reduce recurrence risk in cases where GTR is not possible, while others caution against it due to the potential for malignant transformation, particularly in benign tumors such as CMF [18,19]. Given the aggressive recurrence observed in our case, radiotherapy may have been considered, but the potential risk of sarcomatous transformation requires careful consideration.

Overall, our case highlights the need for individualized treatment approaches based on tumor location, the extent of surgical resection, and potential post-surgical complications. Further studies are required to explore the full spectrum of neurological manifestations associated with CMF, particularly the rare occurrence of seizures, and to better define the role of adjuvant therapies such as radiotherapy in managing these rare tumors.

## Conclusions

This case report highlights a very rare case of skull base chondromyxoid fibroma, which was treated surgically. Our case underlines the necessity of performing a GTR of this benign tumor to ensure minimal risk of recurrence. The involvement of numerous anatomical compartments, including the right temporal pericerebral area, posterior fossa, and suprasellar fossa, determined a challenging surgical treatment that led to the patient undergoing STR.

The unique feature of this case report is represented by the complex clinical presentation of our patient, in particular epileptic seizures. In the end, it is necessary for a patient with skull base CMF to undergo multiple follow-up checkups in order to keep track of the disease progression, ensure a constant quality of life, and to prevent further complications.
